# PRDM9 Drives Evolutionary Erosion of Hotspots in *Mus musculus* through Haplotype-Specific Initiation of Meiotic Recombination

**DOI:** 10.1371/journal.pgen.1004916

**Published:** 2015-01-08

**Authors:** Christopher L. Baker, Shimpei Kajita, Michael Walker, Ruth L. Saxl, Narayanan Raghupathy, Kwangbom Choi, Petko M. Petkov, Kenneth Paigen

**Affiliations:** 1 Center for Genome Dynamics, The Jackson Laboratory, Bar Harbor, Maine, United States of America; 2 Okayama University, Graduate School of Natural Science and Technology, Okayama, Okayama, Japan; Fred Hutchinson Cancer Research Center, United States of America

## Abstract

Meiotic recombination generates new genetic variation and assures the proper segregation of chromosomes in gametes. PRDM9, a zinc finger protein with histone methyltransferase activity, initiates meiotic recombination by binding DNA at recombination hotspots and directing the position of DNA double-strand breaks (DSB). The DSB repair mechanism suggests that hotspots should eventually self-destruct, yet genome-wide recombination levels remain constant, a conundrum known as the hotspot paradox. To test if PRDM9 drives this evolutionary erosion, we measured activity of the *Prdm9* ^Cst^ allele in two *Mus musculus* subspecies, *M.m. castaneus*, in which *Prdm9^Cst^* arose, and *M.m. domesticus*, into which *Prdm9^Cst^* was introduced experimentally. Comparing these two strains, we find that haplotype differences at hotspots lead to qualitative and quantitative changes in PRDM9 binding and activity. Using *Mus spretus* as an outlier, we found most variants affecting PRDM9^Cst^ binding arose and were fixed in *M.m. castaneus*, suppressing hotspot activity. Furthermore, *M.m. castaneus*×*M.m. domesticus* F1 hybrids exhibit novel hotspots, with large haplotype biases in both PRDM9 binding and chromatin modification. These novel hotspots represent sites of historic evolutionary erosion that become activated in hybrids due to crosstalk between one parent's *Prdm9* allele and the opposite parent's chromosome. Together these data support a model where haplotype-specific PRDM9 binding directs biased gene conversion at hotspots, ultimately leading to hotspot erosion.

## Author Summary

Sexually reproducing creatures need to produce germ cells, notably sperm and egg, and do so using a specialized cell division, termed meiosis. A hallmark of meiosis is the process of recombination, in which pieces of maternal and paternal genetic material are exchanged, creating new combinations that are inherited by their progeny. Recombination increases diversity in subsequent generations, facilitating evolution. However, if recombination goes awry, it can lead to genetic disorders and spontaneous abortions in humans. In most mammals the sites of recombination are directed by the enzyme PRDM9 to specific regions on DNA, termed hotspots. For several decades it has been speculated that the process of recombination should lead to the eventual inactivation of hotspots, resulting in the loss of ability to reproduce. The discovery of PRDM9 provided a potential solution to this dilemma when the appearance of new PRDM9 alleles with altered DNA binding specificity would immediately create a new set of hotspots. We have now used the power of mouse genetics and large scale measurements of PRDM9 location and activity to show that this cycle of hotspot loss and recovery does indeed occur over the course of hundreds of thousands of years, and is directed by PRDM9.

## Introduction

Genetic recombination increases variability by facilitating selection for new, favorable combinations of alleles and against deleterious mutations. In many eukaryotes, including humans and mice, recombination is restricted to short (1- to 2-kb) regions known as hotspots [reviewed in 1], [2]. Understanding how hotspots are regulated is fundamental to our understanding of genetics as hotspots determine the pattern of inheritance from one generation to the next.

In most mammals, the meiosis-specific histone methyltransferase, PRDM9 (MGI:2384854), designates the genome-wide position of hotspots through sequence-specific DNA binding of its zinc finger array [3], [4], [5], [6], [7]. Upon binding DNA PRDM9 catalyzes trimethylation of histone H3 at lysine 4 (H3K4me3) and as a result, local nucleosomes are reorganized, creating an extended nucleosome-depleted region (NDR). DNA double-strand breaks (DSBs) are subsequently introduced by the conserved enzyme SPO11 (MGI:1349669) near the NDR [8], [9], [10]. These programmed DSBs are repaired by one of two distinct pathways resulting in either cross-overs (COs), which result in the exchange of flanking DNA, or noncross-overs (NCOs) which do not [10]. Both COs and NCOs can create small gene conversion tracks where the DNA sequence of the activating chromatid is repaired using the homologous partner chromatid as the template [11], [12].

Given this system, all things being equal, one expects hotspot DNA sequences from both parental chromatids should be equally represented in gametes after meiosis. However, multiple studies have identified hotspots where one of the two chromatids has a higher probability of undergoing gene conversion, resulting in frequencies that differ from 50∶50, a phenomenon known as transmission distortion [13], [14], [15], [16], [17]. This observed bias is best explained by the DSB repair model of recombination in which the donor chromatid is used as the template to repair the DNA sequence lost from the active partner in the course of creating the DSB [18]. In cases where this transmission distortion is reported for both COs and NCOs, bias is thought to be established at the initiation of recombination [19], [20]. Biased gene conversion predicts that hotspots should undergo evolutionary erosion whereby the recombination-suppressing (cold) sequence will spread through a population, effectively eroding the hotspot. This leaves the question of how, if hotspots drive themselves to extinction, recombination persists [21].

This hotspot paradox can be thought of as two opposing processes, loss of hotspots through evolutionary erosion, and maintenance of hotspots. A conceptual solution to hotspot maintenance was provided with the discovery of PRDM9, which can overcome the loss of hotspots by undergoing mutations that alter the zinc-finger array of *Prdm9*, creating new alleles that instantly change hotspot positions genome wide. In support of this model, *Prdm9* is under positive selection in both primate and mouse populations, resulting in many different alleles that contain substitutions in the zinc finger domains at each of the three amino acid positions that control DNA binding specificity [22], [23], [24], [25], [26].

Several lines of evidence also suggest that PRDM9 may play a role in hotspot erosion. PRDM9 binds allele-specific consensus motifs at the center of hotspots [8], [9]. Incidentally, polymorphisms undergoing transmission distortion are often found at hotspot centers [11], [13], [15], [17], [27]. At several mouse and human hotspots, single nucleotide polymorphisms (SNPs) showing the largest transmission distortion overlap with predicted PRDM9 binding sites [5], [14], [27], [28]. Furthermore, evidence in primates found that the 13 bp consensus motif associated with one human *PRDM9* allele is disappearing from the human genome faster than the chimpanzee genome [6]. Together, these observations suggest a direct link between PRDM9 binding and evolutionary erosion of hotspots.

Subspecies of mice proved an optimal experimental system to verify these ideas. *Mus musculus* consists of three major subspecies, *M.m. domesticus*, *M.m. castaneus*, and *M.m. musculus*, which diverged from a common ancestor 0.5–1.0 million years ago [29]. Each subspecies possesses its own set of *Prdm9* alleles [22], [23]. If biased gene conversion is driven by PRDM9-directed placement of DSBs, each subspecies should harbor evidence of the evolutionary erosion of its subspecies-specific complement of hotspots within their genomes.

In a previous study we compared the action of two alleles of *Prdm9* in the same genome [8]. These results showed that when the *Prdm9^Cst^* allele, which originated in *M.m. castaneus*, was introduced into the *M. m. domesticus* genome, it activated more hotspots with higher levels of H3K4me3 than did the normally resident *Prdm9^Dom2^* allele. This result suggested that the new combination of *Prdm9* allele and genetic background might lead to increased hotspot number and activity due to the absence of prior evolutionary erosion in the host genome.

We now report evidence confirming this prediction by comparing H3K4me3 levels at hotspots activated by the same *Prdm9^Cst^* allele on two different genetic backgrounds. Additional confirmation was provided by characterizing hotspot usage in *M.m. domesticus*×*M.m. castaneus* and *M.m. domesticus*×*M.m. musculus* F1 hybrids, each heterozygous for different *Prdm9* alleles. Such F1 hybrid mice exhibit a substantial fraction of novel hotspots, not found in either parent. These novel hotspots are shown to be preferentially activated by one PRDM9 protein variant binding to the opposite parental haplotype, for example PRDM9^Cst^ acting on a *M.m. domesticus* chromosome. This sets up haplotype preferences that lead to biased gene conversions. Comparing the two parental hotspot sequences to an outlier, *Mus spretus*, we found that novel hotspots are also sites of historic evolutionary erosion, with increased rates of nucleotide substitution. Further, using a combination of in vitro DNA binding assays and ChIP-seq for PRDM9 itself, we show that hotspot erosion directly affects the ability of PRDM9 to bind hotspot DNA sequences. And finally, we show that historic hotspots are active in intersubspecific hybrids and result in the over transmission of the hotspots-suppressing haplotype to progeny, providing direct evidence that PRDM9 drives this process.

## Results

Recombination hotspots are designated by a complex interplay between the *cis*-acting DNA sequence at hotspots and the *trans-*acting factor that binds to that DNA sequence, namely PRDM9. Both of these features have a large amount of sequence polymorphisms between mouse subspecies. To avoid confusion we have adopted the term alleles to describe sequence variation in *Prdm9* itself and the term haplotypes to describe sequence variation at hotspots themselves.

### The activity of PRDM9 depends on the genetic background

**Figure 1:**
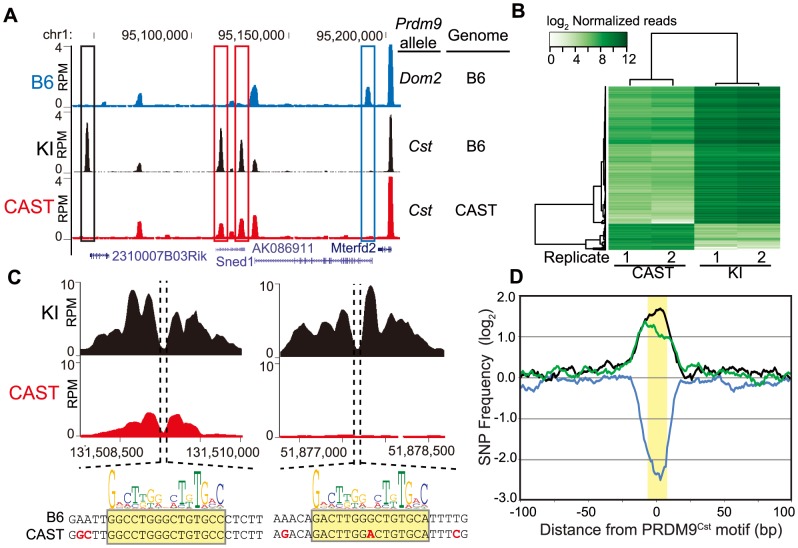
PRDM9 activity is dependent on genetic background. (A) H3K4me3 coverage profile from a representative 150 kb window of chromosome 1. H3K4me3 peaks can be allele specific (red boxes - *Prdm9^Cst^*, blue box - *Prdm9^Dom2^*) or novel (black box - *Prdm9^Cst^* hotspot active on the B6 background). (B) Heat map for shared KI and CAST hotspots with significant differences (n = 2,740, FDR<0.01). (C) Top – H3K4me3 profile for representative PRDM9^Cst^ hotspots with quantitative differences (left) or novel (right). Below - DNA sequence showing SNPs (red) within the hotspot NDR (highlight - PRDM9^Cst^ motif). (D) SNP frequency between the B6 and CAST haplotypes at PRDM9^Cst^ hotspots (10 bp running average normalized to background, yellow highlight - PRDM9^Cst^ motif). Hotspots with differences in H3K4me3 have increased SNP frequency (black –novel hotspots in KI but not CAST, n = 2,035; green – quantitative hotspots from 1B, n = 2,399), while hotspots with similar H3K4me3 level have low SNP frequency (blue – log fold change <0.5 and >−0.5, n = 5,645).

To test for the effects that sequence differences (haplotypes) at hotspots have on PRDM9 activity, we used MNase ChIP-seq for H3K4me3 to develop a genome-wide map of PRDM9-dependent modifications in meiotic cells from juvenile male mice. We first developed H3K4me3 maps for the CAST/EiJ (CAST) strain representing *M.m. castaneus*, which carries the *Prdm9^Cst^* allele characteristic of this subspecies. Comparing these results to our previous H3K4me3 maps from C57BL/6J (B6) and from B6-PRDM9^CAST-KI^/Kpgn (KI), a knock-in strain in which the B6 zinc finger domain of *Prdm9* was replaced by the one from CAST, we find many H3K4me3 peaks are shared among all three strains (n = 54,869, threshold p-value = 0.01), indicating conserved chromatin regulation at sites such as promoters. In addition to shared peaks, we also find putative *Prdm9* allele-specific peaks (Fig. 1A red and blue boxes and S1 Table) and novel peaks that are present in the KI strain only (Fig. 1A, black box). Recent identification of genome-wide DSB hotspots in mice showed that the great majority of breaks, outside of the pseudoautosomal region on the Y-chromosome, are introduced at PRDM9 allele-specific H3K4me3 sites [9], [30]. For the remainder of the manuscript we will refer to PRDM9-directed H3K4me3 peaks as H3K4me3 hotspots.

Of the 24,616 PRDM9^Cst^-dependent H3K4me3 hotspots found in the KI, 9,480 (∼38%) are novel, suggesting that a large fraction of PRDM9^Cst^ H3K4me3 hotspots are uniquely activating on the B6 genome. Moreover, quantifying all H3K4me3 peaks shared between KI and CAST, we found that, in contrast to H3K4me3 found at transcription start sites (TSS), PRDM9-dependent sites were much more likely to differ in H3K4me3 levels between these two strains (S1 Fig.). Among the PRDM9^Cst^-depenent peaks shared between CAST and KI, those with significant differences in H3K4me3 level (false discovery rate <0.01) were much more likely to be higher when present in the B6 background (2,318 out of 2,740, Fig. 1B, S1 Fig.).

### Differences in hotspot H3K4me3 levels are associated with sequence differences within PRDM9 binding sites

We next tested if haplotype differences between the B6 and CAST backgrounds are associated with changes in H3K4me3 levels at hotspots. We first chose two H3K4me3 hotspots, with clear NDRs and PRDM9^Cst^ binding motifs, to represent the two classes of H3K4me3 differences between KI and CAST identified above: one with quantitatively higher H3K4me3 level in the KI strain (chr1 131.51 Mb) and one that was entirely novel to the KI strain (chr1 51.88 Mb) (Fig. 1C). DNA sequencing identified multiple SNPs at each hotspot, both within and near to the PRDM9^Cst^ motif found at the hotspot NDR. These data are in agreement with our previous in vitro mutational analysis of the CAST-activated hotspot *Hlx1* which found that single-nucleotide changes across a minimal 30 bp DNA segment often reduced PRDM9-DNA interaction, even at sites outside the PRDM9^Cst^ motif [4].

We then tested for and confirmed a genome-wide higher frequency of SNPs at these putative PRDM9 DNA-binding sites in the H3K4me3 hotspots that differ in activity between KI and CAST. To do so, we first identified H3K4me3 hotspots for which we could locate a central PRDM9^Cst^ motif and then compared the DNA sequences between the B6 and CAST genomes [31]. We did this for three classes of H3K4me3 hotspots: novel hotspots found only in the KI strain (n = 2,422), hotspots shared between KI and CAST but with significantly different H3K4me3 levels (n = 2,399), and hotspots shared between KI and CAST with similar H3K4me3 levels (log fold change from −0.5 to 0.5, n = 5,645). We used a 2 kb window centered by the PRDM9^Cst^ motif, taking into account the 5′ to 3′ strand orientation of each motif on the chromosome. The number of SNPs found at each nucleotide position was then counted and normalized to the average number of SNPs found across distal 1 kb regions. The SNP frequency across most of the 2 kb region is constant except for large differences in the number of SNPs surrounding the predicted PRDM9 binding sites, dependent on activity class (Fig. 1D). H3K4me3 hotspots unique to the KI strain and those with significant differences in H3K4me3 level both have increased SNPs restricted to the ∼33 base-pairs surrounding the PRDM9^Cst^ motif (black and green lines, Fig. 1D). In contrast, hotspots with similar H3K4me3 activity have reduced SNP frequency indicating the presence of a conserved PRDM9^Cst^ motif (blue line). These data suggest that SNPs found at PRDM9 binding sites influence H3K4me3 levels.

### Intersubspecific crosses create novel hotspots

**Figure 2:**
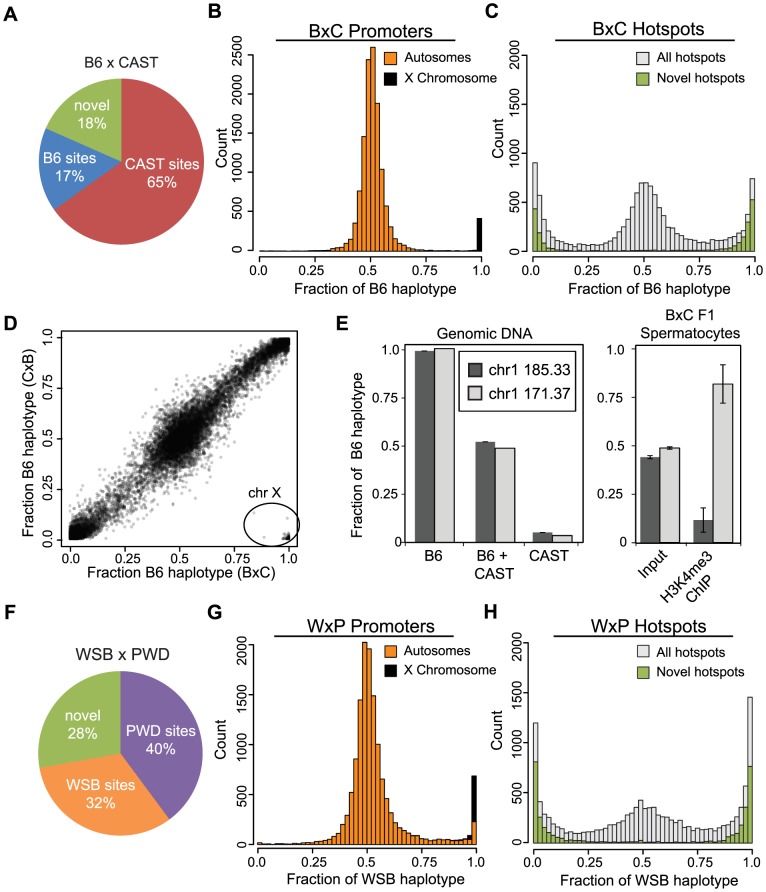
Novel hotspots have biased H3K4me3 modification. (A) F1 hybrid hotspots were classified as B6 or CAST depending on parental origin, or labeled novel. (B) H3K4me3 haplotype ratio for BxC TSS (n = 12,903, orange - autosomes, black - chromosome X). (C) H3K4me3 haplotype ratio for BxC hotspots (grey - all BxC hotspots, n = 12,271; green - novel BxC hotspots, n = 2,298). (D) Scatterplot of haplotype-specific H3K4me3 for hotspots shared between progeny from reciprocal B6 and CAST crosses (n = 10,977, r = 0.978 without X chromosome). 163 hotspots in lower right (circle) are all on the chromosome X. Black shading reflects signal density. (E) Left - Haplotype-specific PCR showing from genomic DNA samples for hotspots chr1 185.33 Mb and chr1 171.37 Mb. Right - Haplotype-specific PCR from spermatocytes before or after enrichment for H3K4me3 (n = 4, error bars - S.D.). (F) The parental identity and fraction of hotpots in WxP F1 hybrids. (G) H3K4me3 haplotype ratio for WxP TSS (n = 15,856, orange - autosomes, black - chromosome X). (H) H3K4me3 haplotype ratio for WxP hotspots (grey - all WxP hotspots, n = 8,360; green - novel WxP hotspots, n = 2,325).

The concept that new hotspots will arise when a PRDM9 allele is faced with a novel genome suggests that novel hotspots should be frequent in F1 hybrids where the *Prdm9* allele from one subspecies now comes in contact with the naïve genome of the other subspecies. To test this, we next performed ChIP-seq for H3K4me3 in F1 hybrid progeny from reciprocal crosses between *M.m. domesticus* and *M.m. castaneus* (Fig. 2 and S2 Fig.). BxC and CxB F1 hybrids have similar numbers of putative PRDM9 H3K4me3 sites compared to either parent, supporting previous evidence showing hotspot numbers do not double in the presence of two alleles (S1 Table) [9]. The F1 hybrid hotspots that overlapped with PRDM9 H3K4me3 peaks present in one of the parents were classified as PRDM9^Cst^- or PRDM9^Dom2^-dependent based on parental origin. The two reciprocal crosses behaved almost identically, with more PRDM9^Cst^-dependent H3K4me3 sites compared to PRDM9^Dom2^-dependent sites (65% versus 17% respectively), an arrangement comparable to previous crosses involving *Prdm9^Dom2^* [9]. The fraction of H3K4me3 hotspots determined by each allele was independent of the direction of the cross (S2A Fig.). Importantly for this study, 18% of all H3K4me3 hotspots in F1 hybrids were not found in either parent, and are classified as novel, fitting our earlier prediction.

### Hotspot H3K4me3 shows large haplotype bias in F1 hybrids

Hotspot erosion predicts that the novel hotspots seen in F1 hybrids arise when the PRDM9 protein variant of one parent preferentially binds to the DNA sequence on the haplotype corresponding to the opposite parent. To determine haplotype specificity we adapted an expectation-maximization based algorithm, originally developed to identify allele-specific gene expression from RNA-seq data, and applied it to quantify haplotype-specific chromatin modifications in the F1 hybrid H3K4me3 ChIP-seq data. For crosses between B6 and CAST mice we define the haplotype ratio as the fraction of H3K4me3 reads that map to the B6 genome divided by the total number of reads for that peak. A ratio of 1 indicates H3K4me3 entirely on the B6 haplotype of the hotspot while a ratio of 0 indicates H3K4me3 entirely on the CAST haplotype.

The majority of H3K4me3 peaks at TSS, representing promoters, do not show strong haplotype specificity (Fig. 2B). An exception is a group of promoters with H3K4me3 on the B6 haplotype, 98% of which are found on the single X chromosome in male mice, which is inherited solely from the B6 mother. In the reciprocal CxB cross, where the X chromosome is inherited from the CAST mother, this observation is reversed and sex-linked promoters are correctly identified as trimethylated on the CAST haplotype (S2B Fig.). These data suggest that the great majority of H3K4me3 modifications at autosomal promoters occur with equal frequency on both parental chromosomes. In addition, the haplotype ratios reported for promoters on the X chromosomes serve as a positive control to demonstrate the specificity of our haplotype analysis.

In contrast to promoters, 42.7% of putative *Prdm9*-dependent hotspots in BxC F1 hybrids show haplotype-specific H3K4 trimethylation (defined as a haplotype ratio <0.25 or >0.75 among hotspots having at least 80 genotype reads, Fig. 2C). As a class, virtually all novel H3K4me3 hotspots exhibit strong haplotype bias (Fig. 2C and S2C, green bars), indicating that one parental chromosome is the primary target for PRDM9-dependent H3K4me3. Genomic imprinting could potentially cause haplotype bias by restricting PRDM9 binding. However, the haplotype ratios of H3K4me3 hotspots are similar in reciprocal B6 and CAST crosses (Fig. 2D), supporting the idea that the bias that does occur arises in *cis* due to local genetic variation.

To assure that haplotype bias at hotspots is not an inadvertent consequence of systematic errors associated with sequencing library preparation or our bioinformatics pipeline, we confirmed these observations for two hotspots using haplotype-specific PCR (Fig. 2E). In spermatocyte chromatin prepared from F1 hybrid animals (‘input’) both haplotypes were represented equally. In contrast, H3K4me3 modification is clearly haplotype-specific (Fig. 2E - left); hotspot chr1 171.37 Mb is predominantly trimethylated on the B6 haplotype while hotspot chr1 185.33 Mb is mostly trimethylated on the CAST haplotype in agreement with our ChIP-seq data (haplotype ratios of 0.96 and 0.01 respectively).

### Haplotype H3K4me3 bias is a general feature of PRDM9 activity

To test if these observations are general features of *Prdm9* alleles we repeated the haplotype-specific analysis by performing H3K4me3 ChIP-seq on WSB/EiJ and PWD/PhJ strains, carrying *Prdm9^Dom3^* and *Prdm9^Msc^* respectively, and on F1 hybrid progeny (WxP) from crosses between them (Fig. 2F–H). In WxP hybrids a ratio near 1 indicates H3K4me3 primarily on WSB haplotypes while a ratio near 0 represents H3K4me3 modification on PWD haplotypes. H3K4me3 hotspots corresponding to the two alleles of *Prdm9* are represented in more equal fractions in the WxP F1 hybrids when compared to the B6 and CAST crosses (Fig. 2F), and a greater proportion (28%) of novel hotspots were also found. For predicted WxP F1 hotspots, 55.8% show haplotype bias, while promoters generally do not (Fig. 2G–H). Furthermore, novel WxP hotspots are again primarily relegated to the extreme ends of the haplotype distribution, suggesting their activation is highly sequence specific only on one parental haplotype.

Collectively, these data show that haplotype-bias and novel hotspots are general features of PRDM9 in at least two different F1 heterozygotes involving four different *Prdm9* alleles, although the number of novel hotspots and fraction of hotspots activated by either parental allele is cross-specific.

### Novel hotspots are preferentially activated by the heterotypic PRDM9 allele

**Figure 3:**
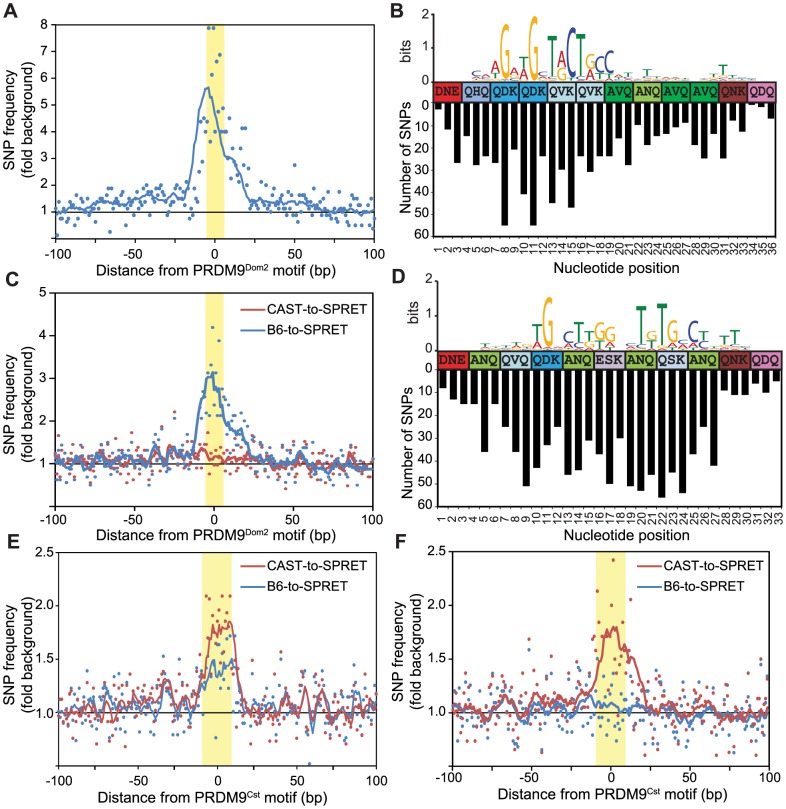
Novel hotspots are the sites of historic hotspot erosion. (A) SNP frequency between B6 and CAST haplotypes for PRDM9^Dom2^-dependent novel hotspots with haplotype ratio <0.2 (n = 810, points - value at each nucleotide, line - 10 bp running average, highlight - PRDM9^Dom2^ motif). (B) Top – CAST haplotype-derived PRDM9^Dom2^ motif for hotspots in *A*. Middle – Amino acid sequence for PRDM9^Dom2^ zinc-finger array positions predicted to contact DNA. Bottom – Number of SNPs identified at each base pair position (background subtracted). (C) SNP frequency between B6 and SPRET (blue) or CAST and SPRET (red) for PRDM9^Dom2^ novel hotspots in *A*. (D) SNP frequency between B6 and CAST haplotypes at hotspots unique to the KI strain (n = 2,035, hotspot represented by black line in Fig. 1D). Top – PRDM9^Cst^ motif derived from the B6 haplotype. Middle – Amino acid sequence for PRDM9^Cst^ zinc-finger array. Bottom – Number of SNPs identified at each base pair position (background subtracted). (E) Similar to *C* except comparing SNP frequency between B6 and SPRET (blue) or CAST and SPRET (red) for hotspots used in *D*. (F) Similar to *E* for PRDM9^Cst^ hotspots quantitatively higher in the KI strain compared to CAST (hotspots from Fig. 1D green line, n = 2,187).

Our model for the appearance of novel hotspots predicts that hotspots which are preferentially trimethylated on one haplotype will be activated by the PRDM9 allele from the opposite strain. For instance, novel hotspots found in BxC F1 hybrids that are PRDM9^Cst^-dependent would have H3K4me3 on the B6 haplotype; otherwise they would be found in the CAST parent. Supporting this prediction, 98.9% of novel H3K4me3 hotspots identified in BxC F1 hybrids with haplotype ratios >0.75 (i.e. primarily modified on the B6 haplotype) are also identified in the KI strain (which is homozygous *Prdm9^Cst^* on the B6 background), proving that these novel hotspots are indeed PRDM9^Cst^–dependent. Reciprocally, hotspots modified on the CAST haplotype are predicted to be activated by PRDM9^Dom2^. Supporting this prediction, only 1.3% of novel BxC hotspots with a haplotype ratio <0.25 (i.e. primarily modified on the CAST haplotype) are found in the KI strain, suggesting these hotspots are activated by PRDM9^Dom2^. Indeed, an extended PRDM9^Dom2^ motif can be derived from BxC novel hotspots with haplotype ratio <0.25 (Fig. 3B) while the PRDM9^Cst^ motif cannot.

### Evolutionary erosion occurs at PRDM9 binding sites

A critical question raised by these data is whether novel hotspots arose by accumulation of hotspot-inactivating mutations in the parental strain or hotspot-activating mutations in the partner strain. To resolve this issue we repeated the positional SNP analysis, performed earlier on H3K4me3 hotspots found in KI and CAST mice (Fig. 1D), this time focusing on BxC F1 novel hotspots predicted to be activated by PRDM9^Dom2^ (810 H3K4me3 hotspots identified with PRDM9^Dom2^ motifs and haplotype ratios <0.25). SNP frequencies between the B6 and CAST genomes at these hotspots are again increased specifically across the ∼36 base pairs predicted to bind PRDM9^Dom2^ (Fig. 3A and 3B). Moreover, the greatest numbers of SNPs have accumulated at nucleotide positions that align with the least degenerate bases in the PRDM9^Dom2^ motif (Fig. 3B). This observation is important as this PRDM9^Dom2^ motif was derived by comparing the sequences of the CAST haplotype at these hotspots, while the SNP analysis counts the number of differences between B6 and CAST haplotypes in the same hotspot. The fact that these two measurements align likely indicates their functional significance to PRDM9^Dom2^ binding.

To infer ancestral haplotype we compared the B6 and CAST sequences at these PRDM9^Dom2^-dependent BxC hotspots to the same loci in the outgroup *Mus spretus* (SPRET), which diverged from *Mus musculus* ∼1.5–2 million years ago [29]. Among these PRDM9^Dom2^ hotspots, the CAST genome shares more sequence similarity with SPRET than does the B6 genome, indicating that the CAST haplotype is most often closer to the ancestral sequence (Fig. 3C). We conclude that mutations diminishing the H3K4me3 levels of these novel hotspots were fixed by PRDM9-driven haplotype selection and subsequent biased gene conversion in the B6 lineage, where PRDM9^Dom2^ initiates recombination.

We repeated this evolutionary analysis, splitting PRDM9^Cst^-dependent hotspots into two groups: those that are unique to the KI strain and reappeared as novel in BxC F1 hybrids with haplotype ratio >0.75 (black line, Fig. 1D), and those with quantitative differences between KI and CAST strains (green line, Fig. 1D). Using the first group we derived an extended motif for PRDM9^Cst^ and aligned it to the zinc finger motif (Fig. 3D top). Similar to PRDM9^Dom2^ novel hotspots, SNPs between B6 and CAST strains at these PRDM9^Cst^ hotspots align with important nucleotides in the motif (Fig. 3D bottom). Next we compared both B6 and CAST haplotypes to *M. spretus* at these hotspots, finding an appreciably higher SNP frequency between CAST and SPRET (Fig. 3E). Surprisingly, there is also an increased SNP frequency between B6 and SPRET at these PRDM9^Cst^ hotspots. We extended this evolutionary analysis to the second group of PRDM9^Cst^ hotspots that are quantitatively higher in the KI compared to CAST (green line Fig. 1D). These data again show that the SNP frequency between CAST and SPRET is increased specifically at the PRDM9^Cst^ binding sites, without any difference in SNP frequency between B6 and SPRET (Fig. 3F). These data indicate that the B6 haplotypes at PRDM9^Cst^ hotspots are most often closer to the ancestral sequence, and that mutations diminishing hotspot activity are accumulating specifically in the *M.m. castaneus* lineage where *Prdm9^Cst^* is active.

Positional SNP analysis was repeated on H3K4me3 hotspots from WxP F1 hybrids. Clustering based on haplotype-specific H3K4me3 ChIP-seq signal from WxP F1 hybrids divided these putative WxP hotspots into three main groups (S3A Fig.). Groups 1 and 3 represent hotspots primarily H3K4 trimethylated on either the WSB or PWD haplotype respectively, while group 2 represents hotspots with approximately equal modification. Haplotype-specific hotspots have the highest number of SNPs specifically centered at the NDR where PRDM9 binds (S3B Fig.).

Together, these observations demonstrate that the novel hotspots found in F1 hybrids correspond to sites of historical evolutionary erosion in one of the parental strains.

### PRDM9 shows preferential binding in vitro

**Figure 4:**
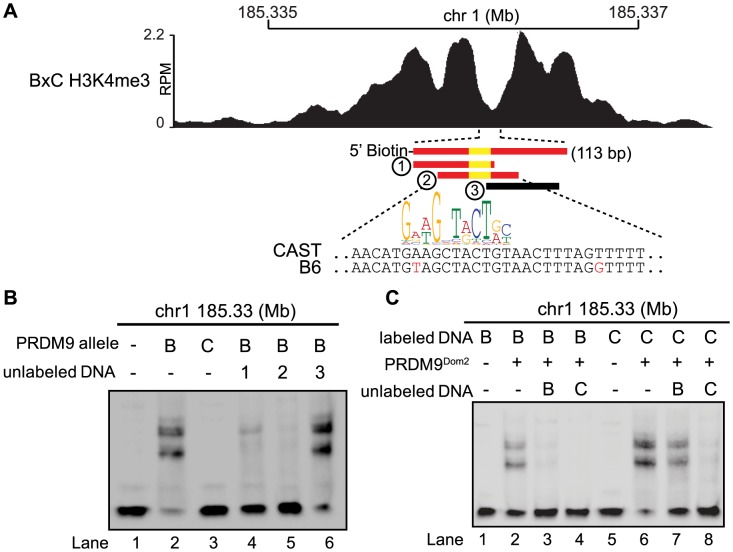
Hotspot haplotype influences PRDM9 binding. (A) H3K4me3 coverage profile from BxC F1 hybrid showing DNA sequences for both B6 and CAST haplotypes and PRDM9^Dom2^ motif (red bars – sequences bound by PRDM9^Dom2^, black bars - nonbinding sequences, yellow - PRDM9^Dom2^ motif). (B) EMSA assay shows specific binding of PRDM9^Dom2^ at the novel hotspot chr1 185.33 Mb (lanes 2 vs. 3). Unlabeled DNA oligos 1 and 2, both containing the PRDM9^Dom2^ consensus motif, can compete for binding (Lanes 4 and 5). All lanes contain labeled DNA of the CAST haplotype otherwise the composition of the binding reactions is shown above the blot (B – PRDM9^Dom2^, C – PRDM9^Cst^). (C) EMSA assay shows PRDM9^Dom2^ can bind to both B6 and CAST haplotypes (Lanes 2 and 6) although the CAST haplotype is preferentially bound under conditions of competition (Lanes 7 and 8, B - B6 haplotype, C - CAST haplotype).

To test if haplotype-bias predicts which PRDM9 protein variant can bind novel hotspots we directly measured protein binding in vitro using a DNA electrophoretic mobility shift assay [4]. The novel hotspot at chr1 185.33 Mb has a haplotype ratio of 0.01, suggesting it is activated by PRDM9^Dom2^ preferentially on the CAST haplotype. In vitro binding confirmed that PRDM9^Dom2^ specifically binds the CAST haplotype, while PRDM9^Cst^ is unable to do so (Fig. 4A and B). This observation was repeated with another novel hotspot at chr1 158.65 Mb activated on the CAST haplotype, showing PRDM9^Dom2^-specific binding (haplotype ratio 0.01, S4 Fig.). Confirmation of the inverse scenario was obtained at two other hotspots, chr1 171.37 Mb and chr1 176.46 Mb. We previously showed that these are PRDM9^Cst^-dependent hotspots activated in the KI strain, and we now report that they appear as novel hotspots in BxC F1 hybrids with most H3K4me3 on the B6 haplotype (haplotype ratios 0.96 and 0.98 respectively). In vitro binding confirmed that PRDM9^Cst^ but not PRDM9^Dom2^ binds these two hotspots [8].

Next we compared the ability of PRDM9 to bind each haplotype individually and for the two haplotypes to compete with each other for binding. Although the two PRDM9^Dom2^-activated hotspots, chr1 185.33 Mb and chr1 158.65 Mb, have SNP differences within the PRDM9 binding site, both B6 and CAST haplotypes still have relatively good matches to the PRDM9^Dom2^ motif (Fig. 4A and S4A). Nevertheless, PRDM9^Dom2^ binds a greater fraction of the CAST haplotype at each hotspot (Fig. 4C lane 6 vs. lane 2, S4D lane 3 vs. lane 8). At hotspot chr1 185.33 Mb, the CAST haplotype completely out competes the B6 haplotype for PRDM9^Dom2^ binding (Fig. 4C lane 4), while the opposite is not true; the B6 haplotype is a poor competitor with the CAST haplotype for PRDM9^Dom2^ binding (Fig. 4C lane 7). These data show that for novel hotspot chr1 185.33 Mb the preferential PRDM9^Dom2^ binding in vitro reflects the haplotype preference in H3K4me3 level observed in F1 hybrids.

### Evolutionary erosion affects PRDM9 binding in vivo

**Figure 5:**
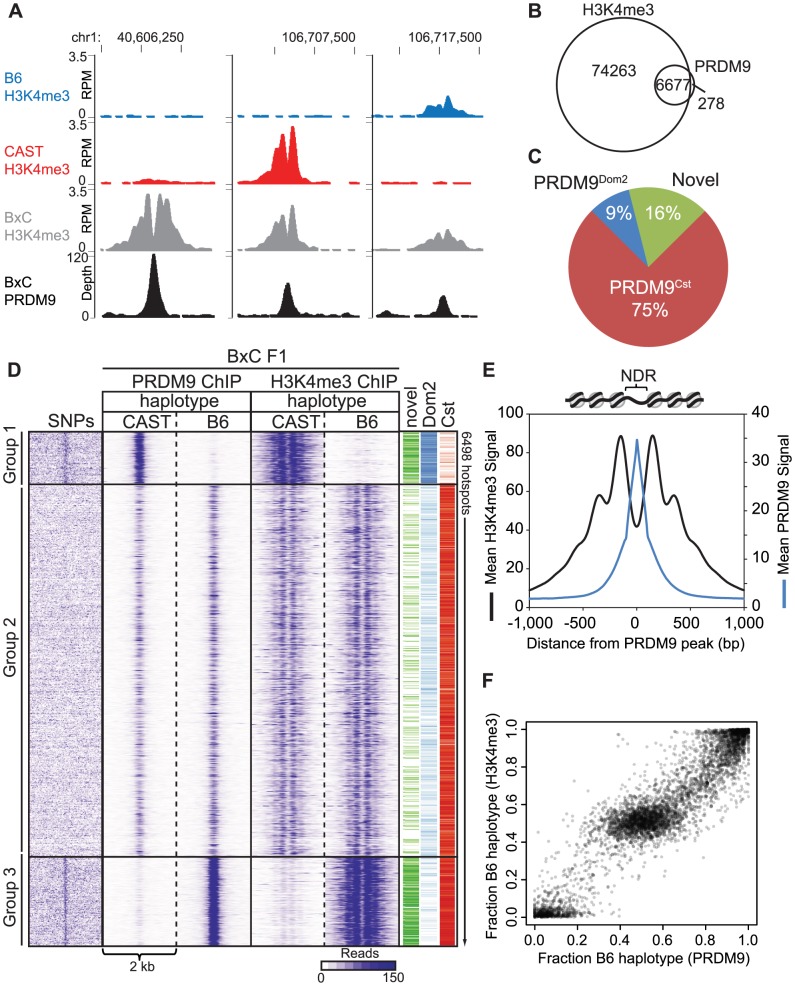
PRDM9 binding shows large haplotype bias in vivo. (A) Coverage profile for several representative hotspots for both PRDM9 ChIP-seq and H3K4me3 ChIP-seq in BxC F1 hybrids. (B) 96% of BxC F1 PRDM9 peaks overlap with BxC F1 H3K4me3. (C) Parental origin of BxC PRDM9 peaks. (D) Haplotype-specific heat map of ChIP-seq signal for PRDM9 and H3K4me3 from BxC F1 hybrids. Read densities from a 2 kb window centered on the summit of PRDM9 peaks were clustered. SNPs density between B6 and CAST strains across the same 2 kb were arranged in the order of hotspots. The activating PRDM9 protein variant, and whether the hotspot was identified as novel, is indicated by color bar on the right. (E) Aggregation plots of PRDM9 and H3K4me3 signal. (F) Scatterplot of haplotype-specific PRDM9 binding and haplotype-specific H3K4me3.

In principle, H3K4me3 ChIP-seq measurements of hotspot activity do not distinguish whether hotspot erosion affects the initial ability of PRDM9 to bind DNA or its subsequent ability to modify nucleosomes. To confirm that nucleotide variants found at hotspots do influence PRDM9 binding in vivo, we measured PRDM9-DNA interaction directly using ChIP against PRDM9 itself in BxC F1 hybrids (Fig. 5). Our N-terminal directed PRDM9 antibody recognizes both PRDM9^Dom2^ and PRDM9^Cst^ (S5A Fig.). While we recover fewer overall BxC PRDM9 ChIP-seq peaks than BxC H3K4me3 ChIP-seq peaks, 96% of PRDM9 ChIP-seq peaks overlap H3K4me3 peaks (Fig. 5B), and represent hotspots with the highest levels of H3K4me3 (S5B Fig.). Importantly, the summits of the PRDM9 peaks fall exactly within the central NDRs of H3K4me3 ChIP-seq signal (Fig. 5A, D, and E). Assigning PRDM9 peaks based on parental origin of the H3K4me3 signal, we can again detect PRDM9^Cst^- and PRDM9^Dom2^-specific and novel hotspots (Fig. 5A and C).

We next wanted to determine the extent to which the haplotype-specific H3K4me3 found at hotspots in F1 hybrids is driven by haplotype-biased DNA binding by PRDM9. F1 hybrid BxC PRDM9 and H3K4me3 ChIP-seq reads were remapped to both the B6 and CAST genomes separately using the methodology of Huang et al. [32] to split the data into parental haplotypes. Sequencing read densities were extracted for 2 kb regions centered on the PRDM9 ChIP-seq summits and subjected to cluster analysis resulting in three main hotspot haplotype categories: group 1 shows preferential PRDM9 binding and H3K4me3 on the CAST genome, group 3 shows preferential PRDM9 binding and H3K4me3 on the B6 genome, and group 2 shows equal binding and equal H3K4me3 (Fig. 5D). Analyzing SNP densities across the same 2 kb hotspot intervals showed that, in contrast to group 2, groups 1 and 3 with high haplotype-specificity also have high SNP density within their NDRs were PRDM9 binds. In addition, there is a strong correlation between the quantitative level of haplotype bias found at individual hotspots estimated from PRDM9 binding and from H3K4me3 levels (Fig. 5F).

Together, these data show that haplotype-specific PRDM9 binding leads to haplotype-specific H3K4me3, and hotspots with the largest haplotype bias are enriched for SNPs specifically located at PRDM9 binding sites.

### Haplotype-specific PRDM9 binding drives transmission distortion at hotspot centers

**Figure 6:**
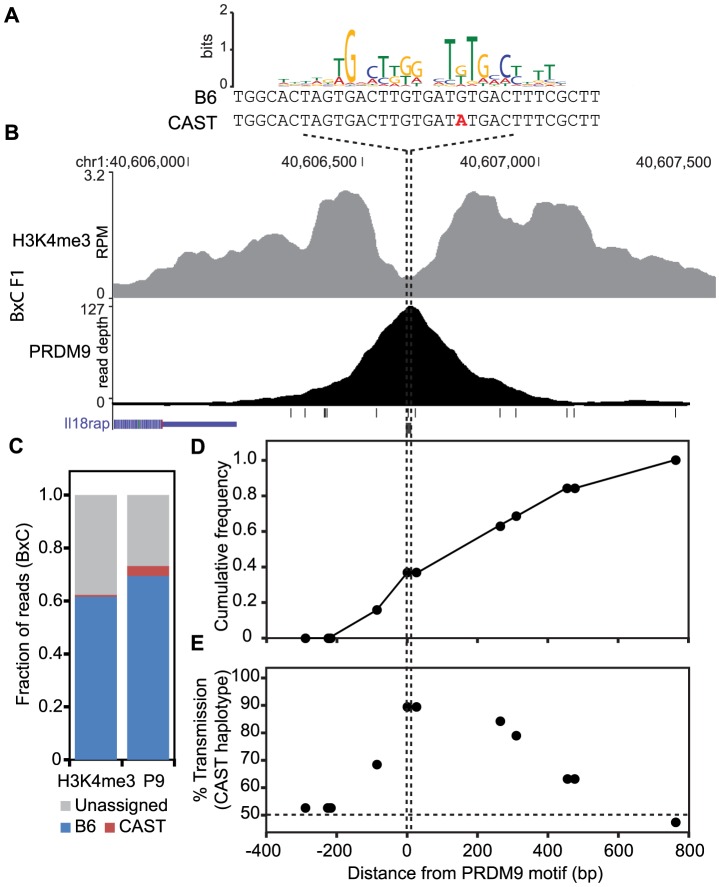
The novel hotspot at chr1 40.6 Mb, activated by PRDM9^Cst^ on the B6 haplotype, shows transmission distortion to the CAST haplotype. (A) PRDM9^Cst^ motif aligned to the DNA sequence for both B6 and CAST haplotypes at the peak of PRDM9 ChIP-seq signal (B) PRDM9 and H3K4me3 coverage profile at chr1 40.6 Mb (black lines – SNP positions between B6 and CAST, black box – PRDM9^Cst^ motif). (C) Proportion of mapped reads to each haplotype (unassigned reads lack informative SNPs). (D) Cumulative frequency of recombination crossovers at hotspot chr1 40.6 Mb (n = 19 mice). (E) Transmission distortion at hotspot chr1 40.6 Mb favors the CAST haplotype at the PRDM9 binding site.

If haplotype-specific PRDM9 binding leads to biased gene conversion, progeny animals should show transmission distortion towards the unbound haplotype. To confirm that this is indeed the case, we tested for biased gene conversion at a novel H3K4me3 hotspot. Previously, crossover positions were mapped for crosses between B6 and CAST animals [17], [33]. We identified a moderately active recombination interval of ∼50 kb on chromosome 1 at 40.60 Mb, with a sex averaged recombination rate of 0.52 cM. This interval also contained overlapping novel PRDM9 and H3K4me3 ChIP-seq peaks in BxC F1 progeny (Fig. 6B). The summit of the PRDM9 peak aligns with a PRDM9^Cst^ motif containing a single SNP between B6 and CAST haplotypes (Fig. 6A). Haplotype analysis of DNA from both PRDM9 and H3K4me3 ChIP-seq showed that 94.8% and 98.9% of all assignable reads map to the B6 haplotype (Fig. 6C). Recombinant progeny were sequenced to determine CO positions (n = 19). Similar to previous observations [8], all COs are constrained to the genomic region within the range of H3K4me3 modification (Fig. 6D). Importantly, there is a ∼90% over transmission of the CAST haplotype to recombinant progeny, specifically at SNPs within the PRDM9 binding site (P<0.0146, Fisher exact test, Fig. 6E). These data show that at this novel hotspot, activated by PRDM9^Cst^, DSBs are introduced on the hot B6 haplotype and preferentially repaired using the cold CAST haplotype, leading to biased gene conversion. These data confirm the hypothesis that biased PRDM9 binding leads to biased initiation of recombination, which ultimately results in hotspot erosion.

## Discussion

We tested the mechanisms of evolutionary erosion of hotspot activity by examining how various genetic backgrounds influence recombination hotspot utilization, PRDM9-dependent H3K4me3 activity, and downstream meiotic recombination. We did this by coupling the power of mouse genetics with molecular assays, primarily ChIP-seq for H3K4me3, the chromatin mark catalyzed by PRDM9, ChIP-seq for PRDM9 itself, and in vitro binding assays. This allowed us to control independently the identities of *Prdm9* alleles and the parental haplotypes of hotspot sequences. We found that both PRDM9 binding and hotspot H3K4me3 show extensive haplotype preferences as a consequence of local sequence polymorphisms. Introducing an allele of *Prdm9* into an evolutionary distant mouse subspecies, either through traditional crosses to produce F1 hybrids or by directed genome editing, resulted in the activation of thousands of novel hotspots. In crosses, these novel hotspots exhibit biased gene conversion driven by preferential hotspot activation through haplotype-specific PRDM9 binding. Using *Mus spretus* as an outlier, we found a considerably increased rate of base-pair substitution at predicted PRDM9 binding sites in the subspecies with which an allele is historically associated. We conclude that novel hotspots represent sites of historic evolutionary erosion.

### Haplotype at PRDM9 binding sites regulates hotspot activation

When a sequence-specific binding site undergoing epigenetic modification is functionally important and hence under evolutionary constraint, one would expect that homologous chromosomes from each parent would be subject to equal modification. In the data presented here, this type of haplotype-independent chromatin modification is found at most gene promoters (Fig. 2B, 2G, and S2B). This observation is not particularly surprising given that the basic meiotic program, established through gene expression, probably differs little from one mouse subspecies to the next. This is in contrast to the strong haplotype biases seen for H3K4me3 hotspots in F1 hybrids (Fig. 2C, 2H, and S2C). Considering the large *cis* differences at the same hotspots in different *Mus musculus* subspecies, there seems to be little evolutionary pressure to conserve PRDM9 binding sequences. Haplotype bias was observed for four *Prdm9* alleles in two crosses, suggesting that bias is a general feature of PRDM9's role in hotspot activation.

Although haplotype-specific chromatin modification has been reported in mouse and humans in the context of gene regulation, these events are generally the exception rather than the rule. Heinz et al. [34] examined binding of several transcription factors and chromatin marks in two strains of *M.m. domesticus*, and their F1 progeny, and found that less than 1% of sites showed strain-specific binding. Additionally three recent reports [35], [36], [37] found heritable, haplotype-specific transcription factor binding and chromatin modification at enhancers in humans, often associated with motif-disrupting SNPs; however, it is not clear the extent to which these events result in allele-specific phenotypes. What sets the haplotype-specific bias reported here apart is the direct influence it has on heritable gene flow in current as well as in future generations.

### H3K4me3 at hotspots and the mechanism of recombination

Evidence presented here, showing that PRDM9 preferentially binds only one homolog at novel hotspots (Figs. 3–5), reveals several features about the mechanisms of recombination. Homologous chromosomes chromatids pair during meiotic prophase I in a process called synapsis. In the process, homologous hotspots somehow find each other in what appears to be a vast sea of DNA, which raises the question of how they manage to do so. One possible explanation could be that PRDM9 binding occurs on both homologs and that protein binding between PRDM9 molecules would provide a bridging mechanism for hotspot homolog recognition. However, as attractive as this hypothesis may be, our present data appear to negate it. There are numerous hotpots where PRDM9 appears able to bind to only one homolog, but that undergo normal recombination, making it unlikely that PRDM9's physical presence on both haplotypes contributes to how hotspots find their partners. Additionally, because haplotype-specific PRDM9 binding results in haplotype-specific H3K4me3 marks (Fig. 5), it is unlikely that H3K4me3 modification of the donor chromatid is required for downstream DNA repair steps such as homology search, strand invasion, or Holliday junction formation.

We previously presented data showing that the average DSB signal is centered over PRDM9-binding sites within NDRs and that, for four hotspots, crossing over is limited to the chromosomal region marked by PRDM9-dependent H3K4me3 [8]. Here, we found that the correlation between H3K4me3 breadth and crossover track length is also observed for a novel hotspot, where H3K4me3 is only on one haplotype. These data suggest that, if our previous hypothesis about the restriction of Holliday junction migration is correct, Holliday junction boundaries might be set by the methylation pattern on the haplotype that undergoes a DSB.

### PRDM9 binding drives evolutionary erosion of hotspots

Determining the strain in which mutations affecting hotspot usage arise and are selected for is critical to understanding hotspot biology. In comparing our B6-Prdm9^CAST-KI^ strain to CAST, most SNP differences resulted in lower PRDM9^Cst^-dependent H3K4me3 activity on the CAST genome. This observation could be explained through two functionally distinct mechanisms. A polymorphism, arising in the B6 lineage, could lead to higher activity or an entirely new hotspot. Such mutations would be hotspot activating only in the context of a *Prdm9^Cst^* allele, but would otherwise be neutral in the B6 background. In contrast, a polymorphism arising in the CAST genome, leading to lower activity or abolishing a hotspot, would be hotspot-suppressing. Both models would account for the activation of novel hotspots in F1 hybrids and have been previously proposed [20], [38], [39].

In total, the data presented here strongly favor a model in which haplotype selection by PRDM9 drives the preservation of hotspot-suppressing mutations through biased gene conversion [19]. Contemporary haplotype biases are a consequence of an evolutionary drive operating against hotspots that is directed by differential PRDM9 binding. This results in the loss of PRDM9 allele-specific binding sequences from the genomes of different *Mus musculus* subspecies due to activity of their resident allele. This same effect has been reported in humans where the binding motif for the human PRDM9^A^ allele is disappearing more quickly from the human lineage compared with chimpanzee [6]. In most cases reported, biased gene conversion is associated with both COs and NCOs, suggesting that bias is established at the initiation of recombination, although transmission distortion may result from preferences in the downstream repair of DSBs as well [24]. This later type of transmission distortion is expected to be independent from PRDM9 activity.

### H3K4me3 haplotype bias predicts sites of biased recombination initiation

Comparing our H3K4me3 data in F1 hybrids with hotspots showing large transmission distortion reveals that haplotype bias in PRDM9 binding results in biased initiation of recombination. A case in point is the *Hlx1* hotspot, which is activated by the *Prdm9^Cst^* allele and initiates ∼2.5 times more often on the B6 chromosome in crosses between B6 and CAST [17]. Here, our analysis of haplotype-specific H3K4me3 in BxC F1 hybrids found that the B6 haplotype at *Hlx1* is also preferentially marked with H3K4me3 (haplotype ratio 0.75), consistent with conversion to the weaker binding CAST haplotype [7]. Similarly, PRDM9^Dom2^ activates the hotspot *A3* in crosses between DBA/2J and A/J mice. *A3* undergoes transmission distortion favoring the hotspot-suppressing A/J haplotype ∼80% of the time [11]. CAST mice have a 13 bp deletion at *A3* that overlaps with the PRDM9^Dom2^ motif. In BxC and CxB F1 hybrids the *A3* hotspot has an H3K4me3 haplotype ratio of 0.93 and 0.98 respectively, which would result in over transmission of the suppressing CAST haplotype. Here we also present additional evidence of transmission distortion at a novel hotspot (Fig. 6), which as a class has the most extreme haplotype biases. Given that nearly half of all hotspots in F1 hybrids have some haplotype bias, these data suggest that in any one cross many hotspots are being subjected to the pressure of evolutionary hotspot erosion.

### Hotspot erosion reveals an evolutionary timeline for PRDM9 alleles

**Figure 7:**
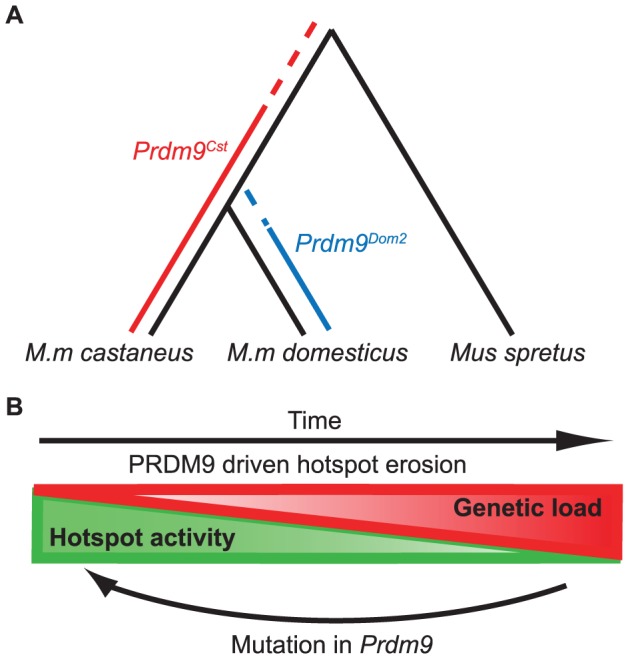
Model for the evolution of *Prdm9* alleles and hotspot erosion. (A) Predicted timeline for the origin of *Prdm9* alleles based on SNP frequency found at hotspots comparing B6 and CAST to SPRET. PRDM9^Cst^ hotspots show an increase in SNPs in the B6 background, suggesting this allele was active in a shared lineage between *M.m. castaneus* and *M.m. domesticus*. PRDM9^Dom2^ hotspots do not have increased SNPs in the CAST background suggesting this allele was never active in *M.m. castaneus*. (B) The PRDM9/hotspot lifecycle. Evolutionary erosion driven by biased PRDM9 initiation of recombination decreases hotspot activity over time at many hotspots in parallel. Mutation of *Prdm9* creates a new binding domain subsequently shifting the genome-wide position of hotspots.

Our SNP analysis at hotspots in the B6 and CAST lineages provides insight into the evolutionary timeline of *Prdm9* alleles (Fig. 7A). PRDM9^Cst^ hotspots that are active in the KI strain, but not the CAST strain, reappear as novel hotspots in BxC F1 hybrids. These hotspots have high levels of polymorphisms in the CAST strain, but surprisingly, they also show a slightly increased level of substitutions in the B6 genome. This suggests that a *Prdm9^Cst^*-like allele arose after *M. musculus* split from *M. spretus* and originally began driving hotspot erosion in the lineage shared between *M.m. domesticus* and *M.m. castaneus*. Later, *M.m. domesticus* diverged and acquired new alleles, ending erosion of PRDM9^Cst^ binding sites in the *M.m. domesticus* lineage. Meanwhile in the *M.m. castaneus* lineage, PRDM9^Cst^-activated hotspots continued to accumulate mutations until they no longer functioned. Moreover, it is likely that the origin of the *Prdm9^Dom2^* allele played a role in the divergence of *Mus musculus* subspecies, given the known role of *Prdm9* in establishing hybrid sterility [40]. This sequence of events is supported by the fact that novel hotspots activated by *Prdm9^Dom2^* in BxC F1 hybrids show no evidence of increased mutation rate in the CAST background, suggesting that the *Prdm9^Dom2^* allele was never operating on the genome of a common ancestor and therefore came later. This timeline also fits with our current understanding that the house mouse originally evolved within the Indian subcontinent, the natural range of *M.m. castaneus* subspecies [22], [23], [29].

### The cyclic evolution of mammalian recombination hotspots

The evolutionary pressure to maintain recombination at hotspots is the result of two alternating processes, operating at different time scales and levels of genetic resolution. These opposing forces give rise to the typical slow-fast behavior of a relaxation oscillator (Fig. 7B). The first process, driven by evolutionary erosion of hotspots, involves a long period of slowly increasing genetic load at PRDM9 binding sites during which hotspots are subject to biased gene conversion favoring hotspot-suppressing haplotypes. Data presented here in mouse and previously in humans [6] suggests that erosion is operating broadly and in parallel at many hotspots genome-wide. A recent prediction for the number of generations required for hotspot erosion showed that once a hotspot inactivating mutation arises, it is likely to become fixed in a population fairly rapidly, within several thousands of generations [27]. This is in marked contrast to the observation reported here, comparing H3K4me3 levels between our KI and CAST strains, that only ∼38% of PRDM9^Cst^-dependent hotspots have lost activity in the CAST lineage over the 1–3 million generations (assuming 2–3 generations per year) since *M.m. castaneus* and *M.m. domesticus* diverged. Combining these observations makes it clear that the rate limiting step in hotspot erosion is almost certainly the origin of new mutations. This conclusion again raises the question of whether the process of DNA repair during homologous recombination is intrinsically mutagenic and might contribute to rates of hotspot erosion.

The process of hotspot erosion would be predicted to eventually reach a tipping point when the number of hotspots present in a strain drops below a currently unknown threshold and creates a loss of fitness. This long relaxation of recombination potential would be followed by a short period characterized by creation or selection of a new *Prdm9* allele, causing an instantaneous shift to a new family of hotspots. This model is supported by the properties of the B6-PRDM9^CAST-KI^, knock-in strain, in which the evolutionary clock has effectively been turned back, resulting in increased activity and number of PRDM9^Cst^ activated hotspots on ancestral sequences. Previous theoretical solutions for the hotspot paradox have included models that involve both *cis* and *trans* control of hotspot activation [38]. These models predict that *cis-*polymorphisms will become more prominent in older hotspots. By counting the number of polymorphisms seen in a collection of historically inactive hotspots for two different alleles, this prediction is demonstrated to now be true in mice. This type of dynamic system is predicted to be stable, yet periodic, providing continued maintenance of recombination potential, and has been modeled as an intragenomic conflict between selfish *cis* hotspot sequences and *trans* hotspot activators [41]. This type of system also may provide an explanation for the rapid evolution of *Prdm9* alleles both between and within mammalian species [22], [23], [24], [25], [26], [42], [43], [44].

## Materials and Methods

### Mouse strains and genotyping

C57BL/6J (stock number 000664), CAST/EiJ (stock number 000928), WSB/EiJ (stock number 001145), and PWD/PhJ (stock number 004660) mice were obtained from The Jackson Laboratory, Bar Harbor, USA. PRDM9^Cst^ Knock-in mice (B6-Prdm9^CAST-KI^/Kpgn) were previously described [8], B6;129P2-*Prdm9^tm1Ymat^/J* (stock number 010719) strain was described in [45]. All animal experiments were approved by the Animal Care and Use Committee of The Jackson Laboratory (Summary #04008).

For genotyping recombinant mice at hotspot chr1 40.6 Mb, we used a library of DNA samples stored at −80°C taken from progeny from crosses between B6 and CAST mice (Paigen et al. 2008). Previously the position of recombinationfor 26 individual mice was mapped to a ∼50 kb interval overlapping two putative PRDM9-dependent BxC H3K4me3 peaks, potentially representing two hotspots. The H3K4me3 peak at chr1 40606000–40607500 was the larger of the two, possibly reflecting greater activity. DNA primers were designed to amplify ∼2 kb of genomic DNA sequence around the chr1 40.6 hotspot NDR and predicted PRDM9 binding site for all recombinant mice and B6 and CAST parents. The resulting amplicons were sequenced and the position of crossing-over was identified as a transition from homozygous B6 to a heterozygous state. Primer sequences for PCR and DNA sequencing are listed in S1 Table. Of the initial 26 mice mapped, DNA from 2 samples no longer existed, and 5 other samples did not contain cross-overs at the chr1 40.6 Mb hotspot, reflecting use of the other hotspot ∼6 kb away.

Haplotype-specific PCR was performed using fluorescent based KASP genotyping reagents (LGC Genomics) and haplotype-specific primers (S1 Table) with 45 amplification cycles.

### H3K4me3 chromatin immunoprecipitation

ChIP for H3K4me3 was performed as reported previously [8]. Briefly, spermatocytes were prepared from 12 days post-partum (dpp) male mice, crosslinked using formaldehyde, and the chromatin was sheared using micrococcal nuclease to obtain primarily mononucleosomes. ChIP was performed using a commercially available antibody against H3K4me3 (EMD/Millipore, #07-473) and 5×10^6^ spermatocytes per biological replicate. Each replicate represents 5–6 individual mice. For each genotype H3K4me3 ChIP was performed on 2 biological replicates.

### PRDM9 chromatin immunoprecipitation

Antibody against a cloned fragment of mouse Prdm9 representing amino acids 101–170 was raised in guinea pigs. PRDM9 ChIP-seq was performed using the ChIP method from [46] without kinetic enrichment for single-stranded DNA prior to library preparation and with the following modifications. Testes were collected from 12-dpp mice. After removing the tunica albuginea the mass of tubules were loosened by pulling gently apart using forceps in cold PBS. Tubules were transferred to 10 ml PBS and freshly dissolved paraformaldehyde (SIGMA, #158127) added to a final concentration of 1%. Samples were allowed to crosslink for 10 min at room temperature with rotation followed by addition of 1 ml of 125 mM glycine and incubation for 5 more minutes. Following crosslinking, the tissue was disaggregated using a Dounce homogenizer and filtered through 40 µm mesh (Fisher, #08-771-1) to collect a single-cell suspension. Cells were collected by centrifugation at 900×g at 4°C for 5 minutes, followed by washing with 10 ml PBS. The centrifugation and wash was repeated and the final cell pellets were snap frozen in liquid nitrogen and stored at −80°C until use.

To prepare chromatin, cells were suspended in 1 ml Hypotonic lysis buffer (10 mM Tris-HCL pH 8.0, 1 mM KCl, 1.5 mM MgCl_2_) with 1 mM PMSF and 1 X protease inhibitor cocktail (SIGMA P8849) and incubated for 30 minutes at 4°C with rotation to shear cells. Nuclei were collected by centrifugation at 4°C at 10,000×g for 10 minutes and resuspended in SDS lysis Buffer (1% SDS, 10 mM EDTA, 50 mM Tris-HCl pH 8) containing protease inhibitors. The chromatin was sheared using a Covaris E220 sonicator for 15 minutes. Following sonication the cellular debris was cleared by centrifugation at 12,500×g at 4°C for 10 minutes. The chromatin was diluted 1∶1 in ChIP buffer (0.01% SDS, 1.1% triton X100, 1.2 mM EDTA, 16.7 mM Tris-Cl pH 8.0, 167 mM NaCl) and dialyzed overnight in ChIP buffer with one buffer exchange using Slide-A-Lyzer Dialysis cassette with 10 MWCO (Pierce, PI-66382).

For immunoprecipitation chromatin was added to Protein-A Dynabeads (Invitrogen) preloaded with our lab-generated PRDM9 antibody (S5 Fig.) and incubated at 4°C overnight with rotation. Immune complexes were washed once with Low Salt Wash Buffer (0.1% SDS, 1% Triton X-100, 2 mM EDTA, 20 mM Tris-HCl pH 8, 150 mM NaCl), once with High Salt Wash Buffer (0.1% SDS, 1% Triton X-100, 2 mM EDTA, 20 mM, Tris-HCl, pH 8, 500 mM NaCl), once with LiCl Wash Buffer (0.25MLiCl, 1% IGEPAL-CA630, 1% deoxycholic acid (sodium salt), 1 mM EDTA, 10 mM Tris-HCl pH 8), and twice with TE (10 mM Tris-HCl, 1 mM EDTA pH 8.0). In the last TE wash the beads were moved to a fresh tube to help reduce nonspecific background. Beads were resuspended into 250 µl Elution Buffer (0.1 M NaHCO_3_, 1% SDS) and incubated for 15 minutes at 65°C. To reverse crosslinks the supernatant was transferred to a new tube, supplemented with 200 mM NaCl, and incubated for 5 hours at 65°C. Protein was digested by adding EDTA to 10 mM, Tris pH 6.5 to 40 mM, and 200 µg Protease K (SIGMA, #P4850), and incubating for 1 hour at 45°C. DNA was purified using the Qiagen PCR Clean-up kit (Qiagen).

### High throughput sequencing and data processing

Libraries for high-throughput sequencing were prepared using Bioo Scientific's NEXTflex ChIP-Seq Kit without size selection and a 50 ul, 14 cycle, PCR reaction. Sequencing was performed using the Illumina HiSeq 2500 platform. For initial peak calling raw sequences were aligned to the mouse genome NCBI Build 37 (mm9) using BWA (Li and Durbin 2009) with default settings. The alignments were subsequently filtered to retain only uniquely mapped reads. For H3K4me3 ChIP, peak calling was performed on merged biological replicates using MACS (v.1.4.2) (Zhang et al. 2008a) with input DNA for control and ChIP samples as treatment, setting the p-value to 0.01, and nodup = ‘all’. For PRDM9 ChIP-seq, peak calling was performed using MACS (p-value = 0.00001) and removing duplicate reads. All coverage profiles presented in figures were generated using the UCSC genome browser (smoothing window of 5) with bedgraph files generated from MACS after tag shifting. Quantitative analysis of differential H3K4me3 levels was determined using the R package DiffBind with a false discovery rate <0.01 [47].

### Identifying putative PRDM9 sites

In order to compare H3K4me3 hotspots in F1 hybrid mice we first needed to determine the hotspot parent of origin. We measured genome-wide H3K4me3 in four strains of inbred mice. In addition to our previously reported genome-wide H3K4me3 map for B6 and KI strains [8], we choose three wild-derived inbred lines CAST, WSB, and PWD. Data for B6 and KI samples are available at GEO (accession number GSE52628). These strains represent three subspecies of *Mus musculus*, each containing a different allele of PRDM9. Initial strain-specific statistically significant H3K4me3 intervals (peaks) were called using MACS (p-value = 0.01) on merged biological duplicates. To identify putative PRDM9 binding sites, represented by unique H3K4me3 peaks, we created a list of peaks shared in pair-wise comparisons between any two strains, and between B6 and our KI strain, resulting in 113,116 shared H3K4me3 intervals. This pair-wise comparison excluded two combinations, B6 and WSB, and CAST and PWD, due to evidence of shared recombination hotspots (P.M. Petkov personal communication). For strain- or cross-specific putative PRDM9-directed H3K4me3 sites, peaks were again identified using MACS on merged biological duplicates using the input DNA as control and a more conservative cut-off (p-value = 0.00001). We subtracted the less stringent common shared interval list from more stringent strain-specific intervals (S1 Table).

Several lines of evidence suggest that these unique H3K4me3 regions largely represent PRDM9-dependent sites. Using the filtering method above 100% of the PRDM9^Dom2^ sites identified here overlap with those identified previously [8] and 90% of the PRDM9^Cst^ sites found in the CAST strain overlap with previously determined PRDM9^Cst^ sites found in B6-*Prdm9^CAST-KI^* strain (see manuscript). In addition, these putative PRDM9 sites have H3K4me3-modified nucleosomes organized in a profile previously shown to be the result of PRDM9 binding (Fig. 5E and S3 Fig.). Finally, the motif derived from the nucleosome-depleted regions at the unique CAST hotspots identified here (Fig. 3D) match the one derived previously when expressing *Prdm9^Cst^* in the B6 strain. While these data suggest that the majority of these unique H3K4me3 are determined by the meiosis-specific methyltransferase PRDM9, we cannot rule out other strain-dependent methyltransferase activity either arising from changes in *cis-*regulatory sequence, or different *trans*-acting factors. In addition to the potential false-positive errors outlined above, PRDM9-dependent sites from different alleles of *Prdm9* that are in close proximity might be discarded using our method thus potentially generating false-negatives.

### Electrophoretic mobility shift assay

EMSA was performed as previously described (Billings et al. 2013). 5′ Biotinylated DNA primers (Eurofins MWG Operon) were used for PCR of genomic DNA. Complementary synthetic DNA oligos were designed from both strands and annealed to create unlabeled dsDNA and used at 20× concentration of labeled DNA for competition. All oligo sequences are listed in S2 Table.

### Mapping haplotype-specific H3K4me3 modification and PRDM9 binding

Haplotype-specific binding estimates can be error prone due to reference genome bias, where genetic variations present in data, but not in the reference genome used for analysis can lead to alignment errors and biased allele specific estimates [48]. For instance, the parental strains, B6 and CAST, are highly divergent with over 17 million SNPs and 2.5 million indels. To reduce alignment bias in haplotype-specific estimates from the genetic variations, we created subspecies-specific genomes using Seqnature [49], by incorporating known SNPs and indels [31]. We then created selective diploid genomes; for example for BxC F1 hybrids we would create one for B6 haplotype and one for CAST haplotype, for all H3K4me3 peaks and PRDM9 peaks identified by MACS (p = 0.00001). Biological replicates were merged and mapped to the diploid genomes using Bowtie (v. 0.2.8) [50] with the following settings: -S –best -a –strata -m 5 -v 3. We used EMASE (Expectation-Maximization for Allele-Specific Expression) to probabilistically apportion the multireads present in the diploid alignment and estimate effective allele/haplotype level read counts. Accounting for genetic variation in a diploid model and utilizing multireads probabilistically using EMASE helps improves the accuracy of haplotype specific binding estimates. A separate manuscript formally describing the EMASE methodology and software is in preparation.

For the convergence of EM algorithm, we ran EMASE using tolerance = 0.01 and requiring intervals to be considered for convergence to have a minimum of 1 unique reads (uniquely mapped to one haplotype). Prior to EMASE analysis duplicate reads were removed from the PRDM9 ChIP alignment files. The haplotype ratio for each interval was then calculated using the formula (# of reads Haplotype 1)/(Total number of reads mapped to that interval). Requiring a minimum of 80 reads uniquely mapping to either haplotype decreased noise between biological replicates and was used as a final filter for the merged datasets.

### Generation of haplotype-specific heat maps

Haplotype-specific heat maps were created using a bioinformatic pipeline from previously published tools. To recover parental origin of mapped reads in a unified reference coordinate map we remapped BxC F1 hybrid and WxP F1 hybrid samples to individual pseudogenomes (available online at http://www.csbio.unc.edu/CCstatus/index.py?run=Pseudo) using Bowtie with the same settings describe above. For example the BxC H3K4me3 ChIP-seq fastq file was mapped to both the C57BL6/J and CAST/EiJ pseudogenomes separately. Because the PWD/EiJ strain has not been sequenced, we used the closely related PWK/EiJ pseudogenome for mapping WxP samples, possibly accounting for the WSB bias seen in Fig. 2G. This process results in two read alignment files with different coordinates for mapped reads depending on the number of strain-specific insertions and deletions across each chromosome. The pseudogenomes coordinates were remapped to the *Mus musculus* reference genome (build 37/mm9) using lapels software [32]. The two individual mapped datasets were then merged using suspenders [32]. This allows scoring each read as either deriving from the maternal or paternal chromosome. If the parental origin of any read cannot be determined the read is flagged as unassigned. Reads were split into separate alignment files by parental origin and unassigned reads were discarded. The software seqMiner [51] was used to extract the coverage signals from a 2 kb window centered on the PRDM9 ChIP-seq peak summits with tag extension set at 150 bp for H3K4me3 ChIP-seq and 100 bp PRDM9 ChIP-seq with a wiggle step of 1 bp. seqMiner was also used for Kmeans clustering starting with 3 clusters. Increasing the number of clusters resulted in splitting the main 3 groups reported into smaller groups based on signal intensity (number of reads), and since signal intensity in these heat maps is dependent on being able to determine parental strain, which in turn is dependent on SNP density, these clusters were not considered meaningful and therefore ignored. PRDM9-dependent hotspots found on the X chromosome were removed from the final heat maps. The data table containing the 2 kb coverage profile for all hotspots was exported from seqMiner and visualized using Java TreeView [52]. Aggregation plots for the heat maps were generated by taking the mean coverage value across all hotspots in a group at each base pair.

SNPs were collected from publicly available sequencing databases [31] for the 4 kb intervals surrounding PRDM9 ChIP-seq summits and converted into bed file format. The bed files were converted into bam format using bedtools [53]. seqMiner was used to extract the data table for SNPs turning off the tag-extension option and setting the wiggle step to 10, essentially creating a 10-bp sliding window, centering on the PRDM9 ChIP-seq summits. The value for a single occurrence of a SNP was arbitrarily set to 60 to help equalize the signal to the H3K4me3 and PRDM9 ChIP read depth for easier visualization in Java TreeView. The Aggregation plots in S3 Fig. for the SNP profiles were created by summing the number of SNPs within each group for all hotspots at each base pair and normalizing that value to the first 500 bp of the 2 kb window.

For the BxC heat map in Fig. 5 the PRDM9 hotspots were assigned as novel if they were not found in either B6 or CAST parent. Hotspots were assigned as PRDM9^Cst^-dependent if they were identified in the B6-Prdm9^CAST-KI^/Kpgn (KI) strain. The remaining hotspots were assigned as PRDM9^Dom2^-dependent.

### Motif identification and aggregation plot

We used The MEME Suite (v 4.9.0) for motif discovery and sequence searching [54]. MEME was used to identify allele-specific motifs at novel hotspots in Fig. 3. The width of each motif was set to the predicted length based on the allele-specific number of zinc fingers. For novel hotspots with a haplotype ratio ≤0.2 DNA sequence from the CAST pseudogenome was used for motif discovery to account for any SNP differences with the reference genome. Motifs were aligned to their respective PRDM9 zinc-finger array based on previously published alignments [4], [9].

ChIP-seq tag densities were aggregated using the agg-py script from the Aggregation and Correlation Toolbox with nbins = 200 and radius = 1000 [55].

### Western blot

For PRDM9 western blots whole testis were collected from 12 dpp mice, tunica removed, and homogenized in RIPA buffer supplemented with 1 mM PMSF and 1X protease inhibitor cocktail (SIGMA). Protein was quantified using Bradford Reagent (BioRad) and normalized to equal concentration in SDS loading buffer. Protein electrophoresis was carried out at 150 V for 60 minutes using BioRad mini-protean system with 4–15% precast Tris-Glycine gels. Protein was transferred to PVDF membrane (Millipore/EMD) using wet transfer system at 280 mA for 4 hours. PVDF membrane was dried in methanol and incubated in Tris-buffered saline (TBS) with PRDM9 antibody overnight, washed with TBS four times, incubated with secondary antibody conjugated to horseradish-peroxidase in TBS for 1 hour, before final washes in TBS. Western blot was developed using SuperSignal West Pico chemiluminescent substrate (Pierce).

### Data access

All raw sequencing files, peak files, and haplotype-specific tables are available at the Gene Expression Omnibus under accession number GSE60906.

## Supporting Information

S1 Fig Hotspots show greater genetic background-dependent variability than promoters. (A) MA plot of H3K4me3 levels at all PRDM9^Cst^ H3K4me3 hotspots shared between KI and CAST strains (n = 14,855). Hotspots showing significant differences (n = 2,740, false discovery rate <0.01) are marked in red. Dotted line indicates 2-fold difference. (B) MA plot of H3K4me3 level at peaks overlapping TSS in both KI and CAST strains. (n = 14,268, red dots = FDR<0.01).
(EPS)

S2 Fig Haplotype analysis in CxB F1 hybrids. (A) The fraction and identity of hotpots in CxB F1 hybrids. Hotspots were classified by activating allele depending on parental origin, those not found in either parent are labeled novel. (B) Promoters have little haplotype-specific chromatin modification. Distribution of haplotype ratio for H3K4me3 peaks found at TSS (orange – autosomes, n = 12,872; black - chromosome X, n = 437). Promoters with CAST-specific chromatin modification are primarily found on the X chromosome. (C) CxB hotspots show large haplotype bias in H3K4me3 modification. Distribution of haplotype ratio for unique H3K4me3 peaks in CxB F1 hybrids (grey - all CxB hotspots, n = 11,363; green - novel CxB hotspots, n = 2,137).
(EPS)

S3 Fig Haplotype analysis in WxP F1 hybrids. (A) Heat map of H3K4me3 ChIP-seq signal split by haplotype. Groups were clustered using Kmeans and SNPs were organized based on the order of the hotspots in each cluster. (B) Aggregation plot of each cluster group from C. SNP frequency is greater at hotspots with large haplotype bias.
(EPS)

S4 Fig PRDM9^Dom2^ preferentially binds the CAST haplotype at hotspot chr1 158.65. (A) ChIP-seq coverage profile from BxC F1 hybrid showing H3K4me3 nucleosome signals, the central NDR, and DNA sequences surrounding the PRDM9^Dom2^ binding site for both B6 and CAST haplotypes. The best match for the PRDM9^Dom2^ motif is indicated above the sequence (red bars – sequences bound by PRDM9^Dom2^, black bars - nonbinding sequences, yellow - PRDM9^Dom2^ motif). (B) Haplotype structure of the labeled DNA used for EMSA assay (circles – SNPs, boxes – insertion/deletions). (C) EMSA assay showing PRDM9^Dom2^ specific binding (lanes 2 vs. 3). Only oligo #1, containing the motif, can compete against the full-length labeled oligo for binding (lane 4). All lanes contain labeled 181 bp DNA of the CAST haplotype otherwise the composition of the binding reactions is shown above the blot (B – PRDM9^Dom2^, C – PRDM9^Cst^). (D) EMSA comparing binding between CAST and B6 haplotype. The CAST haplotype results in a larger fraction of labeled DNA being bound by PRDM9^Dom2^ (lanes 3 vs. 8). The composition of the binding reaction is shown above the blot including the haplotypes of labeled and unlabeled DNA (B - B6, C - CAST).
(EPS)

S5 Fig N-terminal antibody recognizes both PRDM9^Dom2^ and PRDM9^Cst^. (A) Western blot of mouse strains with various combinations of *Prdm9* alleles decorated with the PRDM9 antibody (asterisk – nonspecific band). PRDM9 null allele (−/−) is from [45] and was crossed to either B6 or CAST strain to generate hemizygous mice. (B) PRDM9 ChIP-seq recovers hotspots with highest H3K4me3 ChIP-seq signal. Distribution of H3K4me3 activity at BxC hotspots comparing H3K4me3 level at all putative PRDM9-dependent H3K4me3 sites to those found via PRDM9 ChIP-seq.
(EPS)

S1 Table The total number of H3K4me3 peaks identified for each strain and putative PRDM9-dependent peaks after subtracting common peaks (see methods).
(DOCX)

S2 Table Primers and synthetic oligonucleotides used in this study. All primer coordinates are to the nearest Mb (NCBI Build 37).
(DOCX)
