# Supplementary material for: PRDM9 Drives Evolutionary Erosion of Hotspots in Mus musculus through Haplotype-Specific Initiation of Meiotic Recombination
Source: PLoS Genet. 2015 Jan 8;11(1):e1004916. doi: 10.1371/journal.pgen.1004916 (PMC4287450; doi:10.1371/journal.pgen.1004916)
Supplement: S1 Table — The total number of H3K4me3 peaks identified for each strain and putative PRDM9-dependent peaks after subtracting common peaks (see methods). (DOCX) [file pgen.1004916.s006.docx]

| **Strain** | **Total H3K4me3 peaks (1x10^-5^)** | **Putative PRDM9-dependent**  **H3K4me3 sites** |
| --- | --- | --- |
| B6-Prdm9^CAST-KI^ | 84,281 | 26,552 |
| C57BL/6J | 83,572 | 17,444 |
| CAST/EiJ | 71,402 | 16,865 |
| WSB/EiJ | 80,273 | 11,243 |
| PWD/PhJ | 69,706 | 17,114 |
| **Cross** |  |  |
| B6xCAST | 80,940 | 18,135 |
| CASTxB6 | 73,797 | 17,805 |
| WSBxPWD | 71,645 | 17,565 |
