# Supplementary material for: PRDM9 Drives Evolutionary Erosion of Hotspots in Mus musculus through Haplotype-Specific Initiation of Meiotic Recombination
Source: PLoS Genet. 2015 Jan 8;11(1):e1004916. doi: 10.1371/journal.pgen.1004916 (PMC4287450; doi:10.1371/journal.pgen.1004916)
Supplement: S2 Table — Primers and synthetic oligonucleotides used in this study. All primer coordinates are to the nearest Mb (NCBI Build 37). (DOCX) [file pgen.1004916.s007.docx]

|  | Name | Sequence |
| --- | --- | --- |
| Primers for EMSA | chr1_185_F^a^ | TCCCAGGACTATGAGTTTAGAG |
|  | chr1_185_R | TACACTGGGCTTCAGGCTGCC |
|  | chr1_185_1^b^ | TCCCAGGACTATGAGTTTAGAGAGAAAAAAAACTAAAGTTACAGTAGCTTCATGTTG |
|  | chr1_185_2^b^ | AGAGAGAAAAAAAACTAAAGTTACAGTAGCTTCATGTTGTAGATGGGTATATACCGCT |
|  | chr1_185_3^b^ | TCATGTTGTAGATGGGTATATACCGCTCCCAGCATTTACATATGGCAGCCTGAAGCC |
|  | chr1_185_B^b^ | AGAGAGAAAAAAACCTAAAGTTACAGTAGCTACATGTTGTAGATGGGTATATACCGCT |
|  | chr1_158_F^a^ | CTCTGAAACCTGAAACCTCC |
|  | chr1_158_R | CCTCAGTATCCCTGCACTTC |
|  | chr1_158_1^b^ | CGTCTGCTCTCTGTAGGAAGTGCTCCATGGACAGTAGCTTCTGCCTTTCCTT |
|  | chr1_158_2^b^ | AAGTGCTCCATGGACAGTAGCTTCTGCCTTTCCTTCCCAATAGTGCAGTTGGCTTTCTT |
|  | chr1_158_3^b^ | TGCAGTTGGCTTTCTTTCATTTTGCTTTCAGCTCTGCAGAGTCGACCCC |
|  | chr1_158_4^b^ | TCTGCAGAGTCGACCCCAGCCCTCTCCTTTCTGTTGGCAGTAGAGTGCCCGACTAACCCTTCT |
| Primers for haplotype-specific PCR | 185336010_ALG | GAAGGTGACCAAGTTCATGCTACGGGACAGCCTGCCAGAC |
|  | 185336010_ALT | GAAGGTCGGAGTCAACGGATTAATACGGGACAGCCTGCCAGAA |
|  | 185336010_C | GTGTCCCTTCGGTTTGTCTCTCTAA |
|  | 171376324_1 | GAAGGTGACCAAGTTCATGCTAAAATTTGAATGTCACATGAGGTCTCATT |
|  | 171376324_2 | GAAGGTCGGAGTCAACGGATTAATTTGAATGTCACATGAGGTCTCATC |
|  | 171376324_C | GGTTTCTGGAGGGAAATTAAATGT |
| Primers for sequencing | chr1_40_1F | GGGAATGACCACGGAGCTCAG |
|  | chr1_40_2F | GAGAGTTCCCAAGTAGCCTG |
|  | chr1_40_3F | ATATCTCCTTGTTTCCTTGG |
|  | chr1_40_4F | CCCATTTGTTGACAAGCAGC |
|  | chr1_40_1R | TGTTCAAAGGGCATAAACAG |
|  | chr1_40_2R | AAATGCATACCTGTCATCTC |

^a^ - Primers were ordered with 5´ Biotin

^b^ - Reverse compliment was also ordered to anneal for generating dsDNA
